# The key role of the level of ACE2 gene expression in SARS-CoV-2 infection

**DOI:** 10.18632/aging.203181

**Published:** 2021-06-11

**Authors:** Yves Lecarpentier, Alexandre Vallée

**Affiliations:** 1 Centre de Recherche Clinique, Grand Hôpital de l’Est Francilien (GHEF), Meaux, France; 2 Department of Clinical Research and Innovation, Foch Hospital, Suresnes, France

**Keywords:** ACE2, Alzheimer's disease, aging, COVID-19, SARS-COV-2

## Abstract

SARS-CoV-2 more readily affects the elderly, especially as they present co-morbidities. In the COVID-19 pathogeny, ACE2 appears to be the key cell receptor for SARS-CoV-2 to infect humans. The level of ACE2 gene expression influences the susceptibility of contracting SARS-CoV-2. In circumstances in which the ACE2 level is low, the incidence of Covid-19 seems to be fewer. Two clinical patterns illustrate this observation, i. e., in infants and in Alzheimer's disease (AD). Very young children and AD patients get little COVID-19, in part probably due to decreased expression of ACE2. The determination of the nasal level of ACE2 gene expression could provide a useful scale to predict the susceptibility to contract the SARS-CoV-2 infection.

## INTRODUCTION

The pandemic coronavirus disease-2019 (COVID-19) affect millions of individuals and the severe acute respiratory syndrome coronavirus-2 (SARS-CoV-2) has been shown to be responsible for it. The primary target for entry into host cells has been identified to be the multifunctional protein angiotensin converting enzyme-related carboxypeptidase (ACE2) discovered simultaneously by Donoghue et al. and Tipnis et al. [1, 2]. SARS-CoV-2 more readily affects the elderly [3], especially as they present co-morbidities such as type 2 diabetes, hypertension, chronic obstructive pulmonary disease and cancers [4, 5]. Mortality due to Covid-19 is highly associated with elderly [3]. In COVID patients, the ACE2 protein level significantly increases in both alveolar tissue and bronchial epithelium of diabetic patients [6] and this can partly explain the high rate of infectivity of SARS-CoV-2 in these patients. In hypertensive and diabetic patients treated with ACE inhibitors or angiotensin II type-I receptor blockers (ARBs), an upregulation of ACE2 has been reported [7]. This could facilitate the SARS-CoV-2 infection and increase the risk of developing severe and fatal SARS-CoV-2 infection.

In the COVID-19 pathogeny, ACE2 appears to be the key cell receptor for SARS-CoV-2 to infect humans [8–12]. The human coronavirus SARS-CoV-2 binds with its target cells through ACE2, which is largely expressed, particularly in lung, intestine, kidney and blood vessel cells [7]. Thus, SARS-CoV-2 uses its spike protein S1 to enter cells by interacting with the ACE2 receptor on the cell surface membrane. Thus, SARS-CoV-2 acts directly on cells through the ACE2 receptor leading to downregulate the ACE2 expression by binding with the spike (S) viral protein [8, 12, 13]. This decreases the degradation of angiotensin II (Ang-II) into angiotensin1-7 (Ang-(1-7) [14]. ACE2 converts Ang-II to Ang-(1-7) and prevents the effects of the ACE1/angiotensin II axis. Ang-II induces strong vasoconstriction, inflammatory effects, and profibrotic effects, while Ang-(1-7) exhibits antiproliferative, antiapoptotic, and mild vasodilation and protects against various cardiovascular diseases [15].

The question arises whether the level of ACE2 gene expression influences the susceptibility of contracting SARS-CoV-2. Since SARS-CoV-2 infects the elderly especially as they present co-morbidities with high levels of ACE2 [2], it can be hypothesized that in circumstances in which the ACE2 level is low, the incidence of COVID-19 might be fewer. Two clinical patterns make it possible to illustrate this point, i. e., ACE2 level expression in very young infants and in Alzheimer's disease (AD).

## DISCUSSION

### SARS-CoV-2 in infants

In infants compared to adults, numerous studies have highlighted the low rates of SARS-CoV-2 infection [16]. Children account for about 2% of cases of Covid-19 [17]. Children have lower risk of SARS-CoV-2 infection and mortality [18]. Moreover, in the nasal epithelium of the upper airway, a lower ACE2 expression has been recently reported in infants [19]. Importantly, there is an age-dependent ACE2 gene expression in the nasal epithelium which represents the first contact between SARS-CoV-2 and the human body. ACE2 gene expression is lowest in younger infants (aged <10 years), and progressively increases with age in older infants (aged 10-17 years), young adults (aged 18-24 years) and adults (aged > 25 years). The positive and quasi linear relationship between ACE2 gene expression and age appears to be independent of sex and asthma. The lower nasal ACE2 gene expression in children compared to adults may partly explain why SARS-CoV-2 is less prevalent in infants and why they have lower risk of mortality [20].

### SARS-CoV-2 in Alzheimer's disease (AD)

A clinical study has reported that in AD patients with SARS-CoV-2 pneumonia, the duration from hospitalization to discharge has been shown to be shorter and AD-SARS-CoV-2 patients are less likely to report fatigue and present a better prognosis than non-AD SARS-CoV-2 patients [21]. AD patients have a higher clustering onset than non-AD patients. The duration from the onset of symptoms to hospitalization are shorter in AD patients. The role of ACE2 activity in the AD pathophysiology has been reported [22, 23].

In the central nervous system, ACE2 also presents a non-enzymatic function, by hydrolyzing the key peptide amyloid-β which plays a key role in the AD pathogenesis. Substrates for ACE2 include amyloid-β peptides. ACE2 can efficiently hydrolyze Aβ43 to Aβ42, which is further degraded to the less toxic Aβ40 by ACE [24]. Aβ43 represents a highly amyloidogenic Aβ peptide that induces plaque formation in the brain. Importantly, ACE2 activity is significantly reduced in the frontal cortex and hypothalamus of OXYS AD rats and in the mid-frontal cortex of AD patients, and this is associated with increased parenchymal Aβ and Tau levels [25, 26]. ACE2 is reduced in AD in association with increased amyloid-β and Tau [25]. Conversely, ACE2 overexpression reduces the brain Aβ-induced disease or ACE2 activation by diminazene aceturate improves the cognitive performance [27, 28] and ameliorates Aβ-induced inflammatory processes [29, 30]. In ACE2 knockout mice, the cognitive functions decrease [31].

Ang-(1-7) reduces the AD-related disease. Angiotensin-(1-7) administration attenuates AD disease-like neuropathology in rats with streptozotocin-induced diabetes via Mas receptor activation [32]. Intracerebroventricular infusion of Angiotensin-(1-7) ameliorates cognitive impairment and memory Dysfunction in a AD mouse model [33]. Ang-(1-7) level is decreased in the plasma of AD patients [34] and in brain tissue of AD mice. Ang-(1-7) is reduced and there is an inverse relationship between Ang-(1-7) and Tau hyper-phosphorylation in AD animal models [35] which induces neurofibrillary tangles, a histopathological hallmark of AD. Moreover, AVE0991, a nonpeptide analogue of Ang-(1-7) attenuates aging-related neuroinflammation [36]. A novel Angiotensin-(1-7) glycosylated Mas receptor agonist has been used for treatment of inflammation-related memory dysfunction cognitive impairment in a mouse model [37]. In rat neurons, side-chain-oxidized oxysterols upregulate the ACE2 expression level and Mas receptor [38]. The Ang-(1–7) / Mas receptor signaling is deceased in AD patients [23]. Ang (1–7) levels have been found to be reduced in cerebral cortex and hippocampus in an AD sporadic mouse model associated with hyperphosphorylation of Tau [35]. Furthermore, in AD patients, the plasma concentration of Ang-(1–7) is significantly reduced, suggesting that plasma Ang-(1–7) may represent a potential biomarker for AD diagnosis [34]. As the ACE2 gene expression is reduced in AD patients [25], the decrease in ACE2 expression level could protect against SARS-CoV-2 infection in AD and might partly account for the resilience observed in AD- SARS-CoV-2 patients.

Taken together, these studies show that the ACE2 expression level is decreased in AD [39].

## CONCLUSIONS

In conclusion, very young children get little COVID-19, probably partly due to the decreased expression of ACE2 at the nasal level. AD patients without associated co-morbidities seem relatively protected against Sars-CoV-2 partly due to the decreased expression of ACE2 at the cerebral level. The determination of the nasal level of ACE2 gene expression could provide a useful scale to predict the susceptibility to contract the SARS-CoV-2 infection.
